# On the Effects of Emetics in the Young Subject

**Published:** 1846-10

**Authors:** 


*On the effects of Emetics in the Young Subject, by* John B. Beck, M. D., *Prof. of Mat., Med. and Med. Jur. in College of Physicians and Surgeons of New-York.*

From an article entitled as above, in the Sept. No. of the New-York Journal of Medicine, we extract the practical deductions, as follows:

"1. As a general rule we need not be afraid of vomiting the youngest child, provided the means used be mild—such as ipecacuanha, &c. The mere act of vomiting is attended with no danger, while the remedial agency of an emetic is one of great power and value. Besides acting on the stomach, it extend its influence to the mucous membrane lining the pulmonary organs, promoting secretion in the first place, and then aiding in dislodging and ejecting morbid accumulations; accordingly, in pulmonary affections, there is nothing so efficacious.

2. The vomiting induced by the preparations of antimony ought to be resorted to with great caution in very young children, and should never be used except in those cases where a sedative effect is required, and can be borne with safety. Inflammatory excitement ought then always to be present to justify its use in a young child. Where the object is simply to evacuate the stomach, it ought never to be thought of. In such cases as pneumonic inflammation, it may be justifiably and beneficially used. In those cases it will be found, that the system can bear the sedative influence of the article much better than it can in the ordinary conditions of the system. Even here, however, care should be taken not to push the article too far, as dangerous collapse has been known sometimes to be the result.

3. The *continued use* of Tartar Emetic in young subjects cannot be too specially gaurded against. It is in this way, probably, that

*Andral.

it is so apt to prove injurious. A single dose, even though it vomits very freely, may be borne with comparitive impunity, while the repetition of it may keep up nausea and intestinal irritation, so as to cause injurious prostration. This is very likely to happen in cases of a chronic character, like hooping-cough. Although mild emetics are among our best remedies in this disease, and where the subject is old enough, a single emetic of antimony is frequently exceedingly beneficial, yet the repeated use of antimonial emetics, as is too often the case, appears to me to be a great error in practice. It is not indicated by the nature of the symptoms, and violates a great rule which ought always to be observed in the management of chronic cases, and that is, not to break down unnecessarily the strength of the patient.* Again, in ordinary catarrhal affections in children, a good deal of mischief is frequently done by the continued use of expectorant mixtures containing this active article. The Hive Syrup of Dr. Coxe, which is now in every family, and is given on the slightest occasion to infants, without even consulting a physician, has, I am convinced, done a great deal of harm. I say this without wishing to undervalue this preparation. In proper cases it is really a useful article, but persons out of the profession ought to know that its principal efficacy is owing to the quantity of Tartar Emetic which it contains, and that the indiscriminate use of it in cases where mild articles are required, must be injurious.†

4. As the effect of Tartar Emetic on the system cannot always be measured by its emetic operation, even in the adult, this fact ought to serve as a caution againt the too common practice of giving repeated doses of it to produce vomiting in children, when they happen to be narcotized. While it fails to vomit, it may still operate as a poison to the system. In all cases of this kind, the proper method of treatment is, not to push the emetic, but to endeavor to restore the sensibility of the patient, and then sometimes vomiting comes on at once.

5. In using Tartar Emetic in children, especial regard should be had to their constitutions. In those naturally delicate, and especially where the scrofulous diathesis exists, it should never be used if it can be avoided. Prostration is much more apt to ensue in them, and where the article is persisted in for any length of time, is sure to do harm. It is in such constitutions, when laboring under hooping-cough, and where the use of this article has been too long continued, that the baneful effects of it are most strikingly observed.

* Dr. Armstrong says that "it is a most notorious fact, that the hooping-cough is far more fatal in London than in the country; and I believe," he adds, "that this arises from the very free use of antimonials in London." Lectures, p. 248.

† Every ounce of Coxe's Hive Syrup contains one grain of Tartar Emetic. My friend, Dr. M'Cready, has communicated to me the particulars of a case in which a child between four and five years of age, laboring under hooping-cough, manifestly sunk under the too frequent use of this article. The exhibition of it had been continued about eight days, when symptoms of intestinal irritation came on, accompanied with great general prostration, which in a few days ended the child's existence.

6. It is perhaps hardly necessary to say that if Tartar Emetic be an article of such danger, the younger the subject to whom it is given, the more likely it is to do harm. In children under a year, I should say, as a general rule, it ought never to be used. During that period, the powers of life are too feeble to bear so active a remedy, at the same time that all the beneficial effects of an emetic may be gained from the use of ipecacuanha, or even milder means."

